# Oral, gut, and skin microbiota characterization in patients with heart valve disease with or without infective endocarditis: a pilot study

**DOI:** 10.1128/spectrum.01908-25

**Published:** 2026-07-17

**Authors:** Mariachiara Mengoli, Filomena Boccia, Monica Barone, Ferruccio Mele, Lorenzo Bertolino, Ester Elena Della Ratta, Marisa De Feo, Emanuele Durante-Mangoni, Silvia Turroni, Patrizia Brigidi, Rosa Zampino

**Affiliations:** 1 Human Microbiomics Unit, Department of Medical and Surgical Sciences, University of Bologna 9296, Bologna, Italy; 2 Department of Mental and Physical Health and Preventive Medicine, University of Campania “Luigi Vanvitelli” 404341, Naples, Italy; 3 Unit of Infectious & Transplant Medicine, A.O.R.N. Ospedali dei Colli, Monaldi Hospital 92712 , Naples, Italy; 4 Department of Advanced Medical and Surgical Sciences, University of Campania “Luigi Vanvitelli” 217742, Naples, Italy; 5 Cardiac Surgery Unit, University of Campania “Luigi Vanvitelli”, A.O.R.N. Ospedali dei Colli, Monaldi Hospital, Naples, Italy; 6 Department of Precision Medicine, University of Campania “Luigi Vanvitelli” 550379, Naples, Italy; 7 Unit of Microbiome Science and Biotechnology, Department of Pharmacy and Biotechnology, University of Bologna 9296, Bologna, Italy; Cleveland Clinic Lerner Research Institute, Cleveland, Ohio, USA

**Keywords:** human microbiota, infective endocarditis, heart valve

## Abstract

The relationship between host microbiota and infective endocarditis (IE) has been investigated by few studies. We compared the oral, gut, and skin microbiota profiles of patients awaiting heart valve replacement, both with and without IE, and correlated the profiles with the patients’ clinical data, including the etiological agents and antibiotics received. Thirty-six patients were enrolled in a prospective, observational pilot study: 25 with IE (cases) and 11 with non-infective valve disease requiring surgery (controls). Clinical data and oral, fecal, and skin samples were collected before surgical treatment. Microbiota was profiled using 16S rRNA amplicon sequencing. Cases and controls were comparable, except for inflammatory parameters and prevalence of prosthetic valves, which were higher in cases. Causative IE pathogens were *Staphylococcus* spp., *Streptococcus* spp., and *Enterococcus* spp. Compared to controls, cases showed dysbiotic features in their oral, gut, and skin microbiota, including lower diversity in oral and skin communities and overall community depletion. Only a few bacteria were enriched, such as *Oxalobacteraceae* in the oral cavity and *Bacteroides* in the gut. There was low concordance between bacterial changes and blood isolates for most IE-causing pathogens, suggesting that dysbiosis may have effects beyond merely acting as a reservoir for specific pathogens. Dysbiosis may be a relevant yet underexplored factor associated with IE. Further studies are needed to clarify the underlying pathogenetic mechanisms.

**IMPORTANCE:**

The relationship between microbiota alterations and infective endocarditis has been explored in the literature, but the majority of available studies focus only on the oral microbiota. In this prospective observational pilot study, we analyzed three microbial ecosystems (oral, skin, and gut) in patients awaiting heart valve replacement with and without infective endocarditis. We observed dysbiotic features that are potentially associated with infective endocarditis. While these findings are preliminary, they suggest that future studies should investigate whether microbiota-targeted interventions, such as a probiotic regimen, could play a preventive role in patients with established IE risk factors, including valve defects, presence of prosthesis, or a history of prior IE.

## INTRODUCTION

Infective endocarditis (IE) is a life-threatening infection involving heart valves, prostheses, or devices, often with complications due to systemic spread. Cardiac surgery advances and new antibiotic regimens have improved clinicians’ ability to manage the disease, although mortality and morbidity remain high (1–4). IE pathophysiology is a complex thrombo-inflammatory disorder in which vascular damage, bacteremia, and bacterial virulence factors drive the development of endocardial vegetations. Despite a broad range of presentation patterns and severity, the most common causative pathogens of IE belong to a rather limited set of Gram-positive bacteria, including *Staphylococcus* spp., *Streptococcus* spp., and *Enterococcus* spp. (1). These pathogens enter the circulation from sites where they normally reside (e.g., skin, mouth, gut) or may be inoculated into the subject’s bloodstream during an invasive medical procedure. The presence of a damaged structure is likely to provide a favorable environment for the adhesion of circulating bacteria, which is the critical event for vegetation formation (5).

The term “human microbiota” refers to the complex community of microorganisms, primarily bacteria but also archaebacteria, fungi, and viruses, which inhabit the human body, particularly the gut (6). Depending on the body site, these microbial communities exhibit specific compositional characteristics closely related to the unique features of the habitat (and the selective pressures encountered) and contribute to human health in a variety of ways, modulating critical physiological, metabolic, and immunological functions both locally and systemically (7–9). Changes in the human microbiota (i.e., dysbiosis) have been associated with multiple diseases, even at distant sites. These are often associated with increased mucosal permeability, allowing the translocation of microorganisms and/or their components and prompting systemic effects (10–12).

With regard to IE, only a few studies have investigated its correlation with members of the host microbiota, namely the oral and gut microbiota (13–18). In particular, most of these studies have focused on *Streptococcus mutans*, the major dental caries pathogen, which is a common causative agent of IE (13, 14). More recently, pilot studies using next-generation sequencing to profile the oral microbiota of IE patients, patients at risk of IE, and healthy controls have revealed distinct microbial signatures that may represent a risk factor for IE as well as other systemic diseases (15, 17). A possible causal relationship between gut microbiota and IE has been suggested by a Mendelian randomization study, which provided a list of protective and predisposing genera and potential underlying mechanisms (16). Further research in this area is clearly needed.

In an effort to shed light on this topic, we compared the oral, gut, and skin microbiota profiles of patients awaiting heart valve replacement in the presence and absence of IE. For each ecosystem, the microbiota data were correlated with the patients’ clinical data, in particular the etiological agent and the antibiotic treatment.

## MATERIALS AND METHODS

### Study design

This was a prospective, observational, pilot study, conducted by the Unit of Infectious and Transplant Medicine, in collaboration with the Cardiac Surgery Unit, University of Campania “Luigi Vanvitelli,” Monaldi Hospital (Naples, Italy). Patients admitted to either unit who required heart valve replacement, with or without IE, between November 2021 and December 2022 were included in the study. According to the study protocol, 40 patients were to be enrolled before surgical treatment, of whom 30 with definite IE (cases) and 10 with non-infective valve disease requiring surgery (controls). Diagnosis of IE was made according to the current ESC criteria at the time of enrollment (19). Inclusion criteria for cases were age ≥18 years and confirmed microbiological diagnosis based on positive blood cultures. Patients receiving ongoing antibiotic treatment for >14 days were excluded from the study.

### Patient data collection and sampling

For each enrolled patient, the following data were collected: clinical history, previous invasive procedures, comorbidities, physical examination, echocardiographic parameters (native valve, biological/mechanical prosthesis, vegetation characteristics), dietary habits, laboratory tests at enrollment including blood count, renal and liver function tests, C-reactive protein, coagulation tests, and glycemia. Furthermore, microbiological etiology and detailed information on ongoing (days of treatment, type of antibiotics) and previous antibiotic treatment (length of treatment, type of antibiotics, time since last administration) were collected.

All patients were asked to provide oral, fecal, and skin samples for microbiota analysis. Briefly, for oral microbiota, sterile swabs (eSwab, Copan, Murrieta, CA, USA) were applied in rotation to the right cheek mucosa for 10 s, ensuring the patient did not eat or brush their teeth in the prior 2 h. For skin microbiota, the swabs were applied in rotation to the right axillary skin for 10 s, ensuring the patient did not wash and/or wear deodorants in the prior 2 h. For gut microbiota, feces were collected in sterile containers. Rectal swabs (Fecal Swab, Copan), rotated in the rectum for 10 s, were collected from patients who were unable to provide feces samples. The interchangeability of fecal samples and rectal swabs for the assessment of the gut microbiota profile has been previously demonstrated (20). All samples were stored at −80°C immediately after collection and sent on ice every three months to the Unit of Microbiome Science and Biotechnology, University of Bologna (Bologna, Italy) for microbiota analysis.

### Oral, gut, and skin microbiota profiling

#### Microbial DNA extraction

Microbial DNA was extracted according to previously described protocols (21), with some modifications for oral and skin swabs. Briefly, swabs soaked in 1 mL of their specific storage medium were vortexed for 3 min to recover microbial cells. The suspension was centrifuged at 10,000 rpm for 10 min at 4°C, and the pellet was resuspended in 180 μL of lysis buffer containing 100 mg/mL lysozyme. After incubation for 30 min at 37°C, samples were processed as previously described (21) using the DNeasy Blood and Tissue Kit (QIAGEN, Hilden, Germany). DNA quality and quantity were assessed using a NanoDrop ND-1000 spectrophotometer (NanoDrop Technologies, Wilmington, DE, USA).

#### 16S rRNA amplicon sequencing

Library preparation was performed as previously described (21) using the Illumina protocol “16S Metagenomic Sequencing Library Preparation” (Illumina, San Diego, CA, USA). Briefly, the 341F and 785R primers with Illumina adapter overhang sequences were used to amplify the V3-V4 hypervariable regions of the 16S rRNA gene (22). Amplification was performed using the KAPA HiFi Hot Start Ready Mix (Roche, Basel, Switzerland) with the following thermal cycle: 3 min at 95°C, 25 cycles of 30 s at 95°C, 30 s at 55°C, and 30 s at 72°C, and a final 5-min step at 72°C. A magnetic bead-based clean-up system (Agencourt AMPure XP; Beckman Coulter, Brea, CA, USA) was used to purify the amplicons. Using Nextera technology, limited-cycle PCR was performed to prepare the indexed libraries, and a second clean-up step was performed as described above. After pooling the samples to an equimolar concentration of 4 nM, the final library was denatured and diluted to 5 pM with a 20% PhiX control. Sequencing was performed on an Illumina MiSeq platform using a 2 × 250 bp paired-end protocol. Sequence reads were deposited in the Sequence Read Archive (SRA) (project ID, PRJNA1370008).

#### Bioinformatic analysis

Raw sequence data were processed using a pipeline combining PANDAseq (23) and QIIME 2 (24). After filtering for length (minimum/maximum = 350/550 bp) and quality (default criteria), the remaining reads were binned into amplicon sequence variants (ASVs) using the DADA2 pipeline (25). Taxonomic assignment was performed using the VSEARCH algorithm (26) against the SILVA database (May 2019 release) (27). For species-level identification, ASVs of interest (related to bacterial genera of etiological agents) were subjected to BLASTn analysis against the NCBI 16S rRNA reference database (28). Species assignment required ≥99% sequence identity and ≥98% alignment coverage. Where an ASV mapped to multiple species with identical scores, the most likely species assignment was determined by evaluating alignment length, bit score, and biological plausibility. Alpha diversity was assessed using several metrics, such as the number of observed ASVs, the Shannon index, and Faith’s phylogenetic diversity. Beta diversity was estimated by computing weighted and unweighted UniFrac distances, which were used to generate principal coordinates analysis (PCoA) plots.

### Statistical analysis

Due to the exploratory nature of the study and the limited availability of IE patients, no formal sample size calculation was performed. Accordingly, the current sample size reduces the statistical power, particularly for any subgroup analyses, as discussed in the “Study limitations” section.

For host metadata, Mann–Whitney *U* test and the Pearson test were used to assess the statistical significance of differences between 2 and >2 groups for numerical variables. The Bonferroni correction was used to assess each individual comparison. Fisher’s exact test was used for univariate analysis of categorical data. The significance level was set at 5%, and all tests were 2-tailed. All analyses were performed using the statistical software for Windows SPSS 22 (SPSS, Inc., Chicago, IL, USA).

For microbiota, all statistical analyses were performed using R software. PCoA plots were generated using the “vegan” package (v. 2.6-4, https://cran.r-project.org/web/packages/vegan/index.html), and data separation was tested using a permutation test with pseudo-F ratio (“Adonis” function in “vegan”). The Kruskal-Wallis test, followed by post-hoc Wilcoxon tests, was used to assess group differences in alpha diversity and relative taxon abundances. Genus-level heatmaps were generated using the “made4” package (https://bioconductor.org/packages/release/bioc/html/made4.html) and the “heatmap.2” function from the “gplots” package (https://cran.r-project.org/web/packages/gplots/index.html). The Pearson correlation test was used to evaluate associations between genus-level relative abundances and binary variables of patient metadata, such as gender, history of pathologies including chronic obstructive pulmonary disease (COPD), chronic heart failure, peripheral arterial disease, diabetes, cancer, chronic kidney disease, oral pathologies, rheumatic diseases, dementia, and previous IE. For continuous variables, such as C-reactive protein, hematological parameters (i.e., red blood cell count, white blood cell count, eosinophils, neutrophils, platelets, glycated hemoglobin, mean cell volume, creatinine, azotemia, glycemia, prothrombin time, partial thromboplastin time, glutamic oxaloacetic transaminase, glutamate-pyruvate transaminase), and duration of antibiotic treatment, the Kendall correlation test was used. Only statistically significant correlations with absolute Kendall’s tau and Pearson’s *r* ≥ 0.3 were included. *P*-values were adjusted using the Benjamini-Hochberg method. A false discovery rate (FDR) ≤0.05 was considered statistically significant.

## RESULTS  

### Study cohort description

We screened 51 consecutive hospitalized patients in need of heart valve replacement, 40 with IE (cases) and 11 without IE (controls). Among cases, 15 were excluded for various reasons (three denied consent, five had previously been treated with antibiotics for more than 14 days, and seven had negative blood cultures). At the last follow-up (March 2024), of the 25 cases finally included, 11 (44%) had died, while all controls were still alive (Fisher’s exact test, *P* = 0.015). Table 1 shows the general characteristics of the study group. Fourteen patients were women and 22 were men, with a median age of 67.5 years for cases and 66.5 years for controls. The most common comorbidities among cases were ischemic heart disease, liver disease, and a history of cancer, while comorbidities among controls were COPD, diabetes mellitus, and chronic kidney disease. Valves involved in IE were both native and prosthetic, with native valves being significantly prevalent, mainly at aortic and mitral positions. Among controls, all valves to be replaced were native, 10 aortic, and one mitral. IE patients had significantly higher levels of C-reactive protein, white blood cell count and neutrophils, and lower levels of hemoglobin (Mann–Whitney *U* test, *P* < 0.001, *P* = 0.005, *P* < 0.001, *P* = 0.002, respectively).

**TABLE 1:** Baseline characteristics of the study cohort^*a*^^,*c*^

	IE patients	Controls	*P*
Number	25	11	
Age, years	70 (58–75.5)	62 (60–71)	0.261
Male	15 (60)	7 (63.6)	1.00
Chronic heart failure	6 (24.0)	0 (0)	0.148
COPD	4 (16.0)	2 (18.2)	1.00
Diabetes mellitus	6 (24.0)	1 (9.1)	0.4
Ischemic heart disease	8 (32.0)	0 (0)	0.076
Liver disease	7 (28)	0 (0)	0.076
History of cancer	7 (28)	0 (0)	0.076
Chronic kidney disease	6 (24.0)	1 (9.1)	0.4
White blood cells, cells/μL	9,770 (6,931–12,835)	6,250 (4,682.5–6,995)	0.005
Neutrophils, cells/μL	7,430 (4,960–10,385)	3,640 (3,050–4,062.5)	<0.001
Red blood cells, ×10^3^cells/μL	4,030 (3,248–4,680)	4,610 (4,160–4,847)	0.074
Platelets, cells/μL	209,000 (121,000–276,500)	210,000 (166,500–248,200)	0.919
Hemoglobin, g/dL	10.1 (9.2–11.3)	13.1 (12.13–14.13)	0.002
Creatinine, mg/dL	1.1 (0.8–1.35)	0.9 (0.7–1)	0.086
GPT, U/L	18 (11–31.5)	20 (17.7–36.7)	0.261
GOT, U/L	21 (15.5–727)	19 (18–30.7)	0.892
PT-INR	1.32 (1.15–2.04)	1.08 (1.03–1.13)	<0.001
C-reactive protein, mg/dL	6.9 (4.65–10.15)	0.4 (0.4–0.43)	<0.001
Glycemia	98 (89–129)	104 (94.5–111.2)	0.787
Bowel habits			0.796
Normal	15 (60.0)	7 (63.6)	
Constipated	9 (36.0)	4 (36.4)	
Diarrhoeic	1 (4.0)	0 (0)	
Diet			0.524
Various	24 (96.0)	10 (90.9)	
Low-carbohydrate	1 (1.0)	1 (0)	
Involved valve			0.073
Aortic valve	13 (52.0)	10 (90.9)	
Mitral valve	7 (28.0)	1 (9.1)	
Multivalve	5 (20.0)	0 (0)	
Valve subtype			0.031^*b*^
Native valve	14 (56.0)^*b*^	11 (100)	
Bioprosthesis	8 (32.0)	0 (0)	
Mechanical prosthesis	3 (12.0)	0 (0)	

^*a*^ Data are presented as median [range] or number (percentage). Differences between patients with infective endocarditis (IE) and controls were assessed by Mann–Whitney *U* test for numerical variables, while Fisher’s exact test was used for univariate analysis of categorical data. The Bonferroni correction was used to assess each individual comparison.

^*b*^ Native valves were predominant in both groups.

^*c*^ COPD, chronic obstructive pulmonary disease; GOT, glutamic oxaloacetic transaminase; GPT, glutamate pyruvate transaminase; PT-INR, prothrombin time – international normalized ratio.

Eleven *Staphylococcus* strains (belonging to the species *S. auricularis*, *S. lugdunensis*, *S. haemolyticus*, methicillin-resistant/sensitive *S. epidermidis* [MRSE/MSSE], and methicillin-sensitive *S. aureus* [MSSA]), five *Enterococcus faecalis* strains, nine *Streptococcus* strains (belonging to the species *S. gallolyticus*, *S. mutans*, *S. mitis*, *S. sanguinis*, *S. infantarius*, and *S. oralis*), and one *Corynebacterium striatum* strain were isolated from the blood cultures of the 25 cases. Six cases were enrolled at IE diagnosis, before any antibiotic treatment, while 19 cases were on antibiotics for a median of five days. Patients affected by IE due to *S. gallolyticus* and *Enterococcus* spp. were advised to undergo colonoscopy after IE resolution, owing to the close correlation with gut disorders (29, 30). Only six patients underwent this exam at Monaldi Hospital, resulting in two colorectal adenocarcinomas, two adenomas, and two normal findings. Table 2 shows the etiology and antibiotic regimens in IE patients.

**TABLE 2:** Infective endocarditis (IE) etiology and antibiotic treatment in IE patients^*a*^^,^^*b*^

Patient number	Aetiology	Previous antibiotic duration (days)	Antibiotics
2	*S. auricularis,* *C. striatum*	12	D, Cr
5	*S. lugdunensis*	14	D, Cr
6	MRSE	0	
7	MSSE	12	D, A-C, G
10	MRSE	6	A-C, T-X
11	MSSA	9	D, Cz, Cx
12	*S. gallolyticus*	0	–^*c*^
14	*E. faecalis*	0	–
16	*E. faecalis*	10	D, Cr, G for 6 days; D, A-C, L for 4 days
18	*S. mutans*	0	–
23	*S. mitis,* *S. sanguinis*	3	D, A-C
25	*S. gallolyticus*	9	D, A-C
27	*S. infantarius*	5	A-S, G
28	MSSA	10	V, G
29	*S. gallolyticus*	4	A-C
30	MRSE	6	A-C + T
32	*S. haemolyticus*	14	D for 11 days; Tg for 3 days
33	*S. gallolyticus*	0	–
34	*E. faecalis*	3	A-S, G
35	*E. faecalis*	13	D, G
37	*E. faecalis*	5	D, A-C
38	*S. gallolyticus*	7	Do, Cm, Cfl
39	*S. mitis*/*oralis*	1	Cx
40	MSSE	0	–
41	*S. gallolyticus*	4	Cx, G

^*a*^ Antibiotic treatment included empirical and targeted therapy.

^*b*^ A-C, amoxicillin-clavulanic acid; A-S, ampicillin-sulbactam; Cfl, ciprofloxacin; Cm, cefixime; Cr, ceftaroline; Cx, ceftriaxone; Cz, cefazolin; D, daptomycin; Do, doxycycline; G, gentamicin; L, linezolid; MRSE, methicillin-resistant *S. epidermidis*; MSSA, methicillin-sensitive *S. aureus*; MSSE, methicillin-sensitive *S. epidermidis*; T, teicoplanin; Tg, tigecycline; T-X, trimethoprim-sulfamethoxazole; V, vancomycin.

^*c*^ "–" indicates no antibiotic treatment before enrollement.

### Oral, gut, and skin microbiota of patients with heart valve disease with or without infective endocarditis

16S rRNA amplicon sequencing yielded a total of 6,726,667 sequences, with an average of 63,459 per sample (range, 8,142–689,446), which were grouped into 2,263 ASVs. Two oral samples from cases were excluded from subsequent analyses due to low-quality sequences.

**Fig 1:**
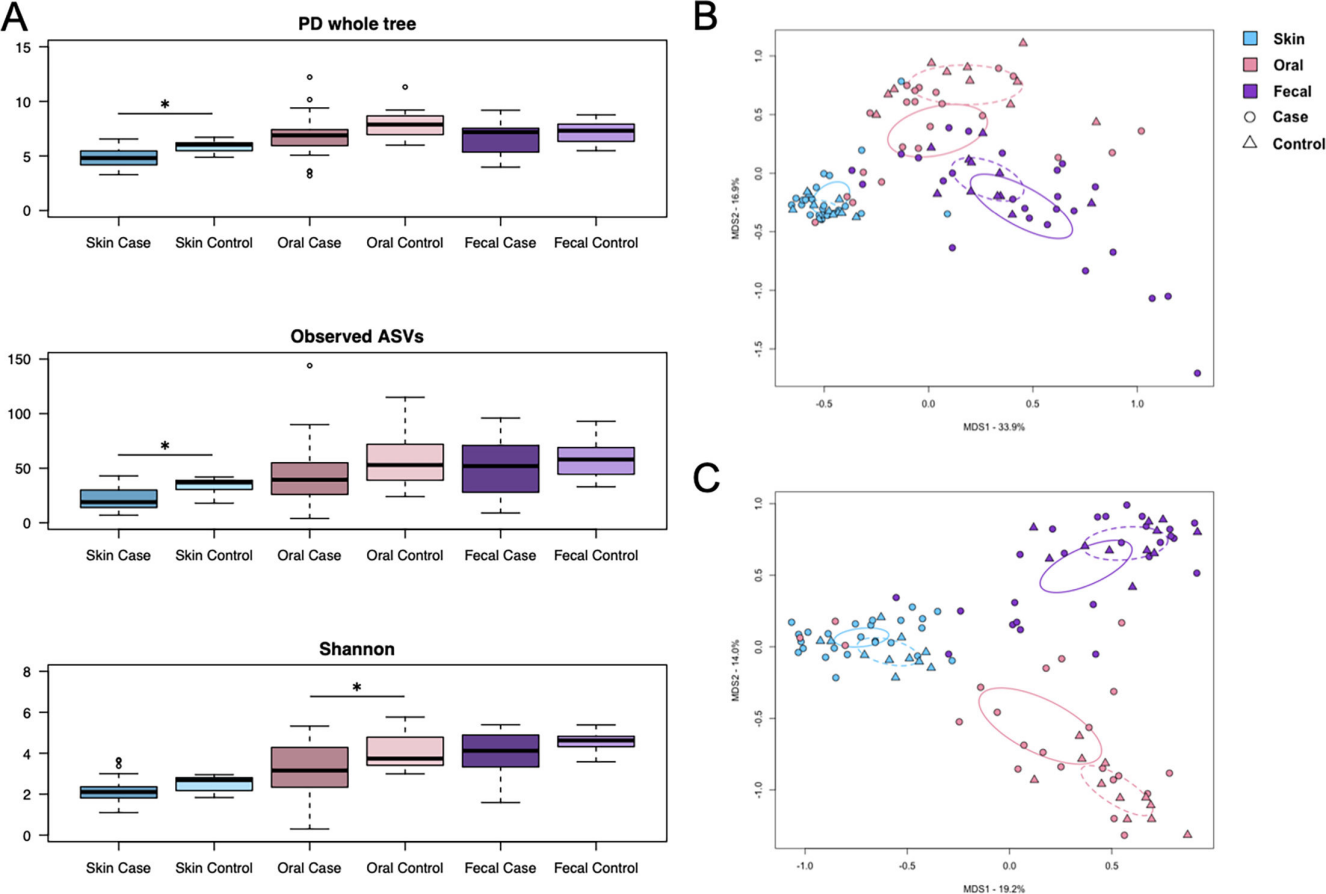
Diversity of the oral, gut, and skin microbiota in patients with heart valve disease with and without infective endocarditis. (**A**) Boxplots showing alpha diversity, estimated using Faith’s phylogenetic diversity (PD whole tree), the number of observed ASVs, and the Shannon index, in the oral, gut, and skin microbiota of cases and controls. Wilcoxon test, *P ≤ 0.05. Principal coordinates analysis (PCoA) based on weighted (**B**) and unweighted UniFrac distances (**C**) between the microbial profiles of the study groups. A significant separation between cases and controls was observed for skin microbiota in the unweighted UniFrac-based PCoA (PERMANOVA, *P* = 0.022).

**Fig 2:**
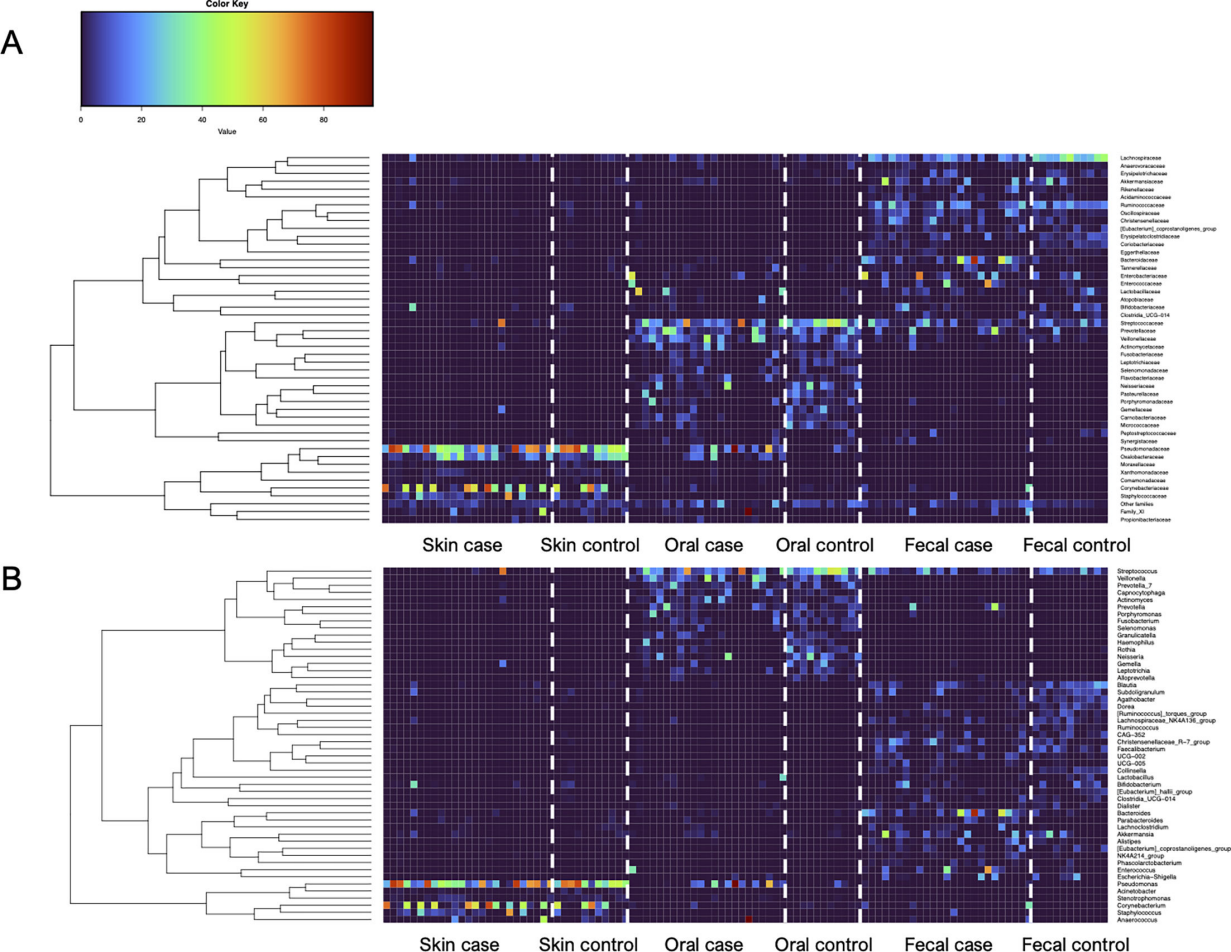
Oral, gut, and skin microbiota signatures of patients with heart valve disease with and without infective endocarditis. Heatmap showing Ward-linkage clustering based on Pearson’s correlation coefficients of the relative abundance of families (**A**) and genera (**B**) in the oral, gut, and skin microbiota of cases and controls. Only taxa with relative abundance ≥1% in at least 20% of samples for each study group are shown. Those with relative abundance <1% were grouped as “Other.” See also Fig. S1 and S2.

Cases showed lower alpha diversity than controls in both skin and oral microbiota (Wilcoxon test, *P* ≤ 0.05), while no differences were found in gut microbiota (Fig. 1A). A significant segregation between cases and controls was also observed for the skin microbiota in the unweighted UniFrac-based PCoA (PERMANOVA, *P* = 0.022) (Fig. 1C). Taxonomically, cases and controls differed in the relative abundance of several taxa, even at the phylum level, in all microbial ecosystems (Fig. 2; Fig. S1 and S2, Table S1). In particular, compared with controls, oral samples from cases showed lower proportions of the phyla Fusobacteriota and Patescibacteria, the families *Erysipelotrichaceae*, *Gemellaceae* (including its genus *Gemella*), *Neisseriaceae* (including *Neisseria*), *Pasteurellaceae* (including *Haemophilus*), *Corynebacteriaceae* (including *Corynebacterium*), *Micrococcaceae* (including *Rothia*), *Porphyromonadaceae* (including *Porphyromonas*), *Fusobacteriaceae* (including *Fusobacterium*), *Leptotrichiaceae* (including *Leptotrichia*) and *Streptococcaceae* (including *Streptococcus*), and the genus *Alloprevotella*, but increased proportions of *Oxalobacteraceae* (Wilcoxon test, *P* ≤ 0.05). The gut microbiota of the cases was enriched in the phylum Bacteroidota with its family *Bacteroidaceae* and the main genus *Bacteroides*, while it was depleted in the families *Coriobacteriaceae* (including its genus *Collinsella*), *Erysipelatoclostridiaceae*, *Lachnospiraceae* (including *Agathobacter*, *Blautia*, *Dorea*, *Lachnospiraceae* NK4A136 group, and *Ruminococcus torques* group), and *Ruminococcaceae* (including *Subdoligranulum*, *Faecalibacterium*, and *Ruminococcus*), as well as the genera *Rothia* and *Prevotella 7* (*P* ≤ 0.05). Finally, skin samples from cases showed lower proportions of the phylum Patescibacteria and the families *Lachnospiraceae*, *Comamonadaceae*, and *Moraxellaceae* and higher proportions of the families *Flavobacteriaceae* and *Streptococcaceae* (including its genus *Streptococcus*) (*P* ≤ 0.05).

Regarding the genera of IE causative microorganisms isolated from blood cultures, as mentioned above, *Streptococcus* was decreased in the oral microbiota and increased in the skin microbiota of cases compared to controls. In particular, ASVs assigned to *S. gallolyticus* were identified in all six cases infected with strains belonging to this species, of whom one in both oral and fecal samples, one in both oral and skin samples, one in both skin and fecal samples, one in skin samples only, and two in fecal samples only. For the other genera isolated, *Staphylococcus* and *Enterococcus*, no differences were found in any of the ecosystems.

**Fig 3:**
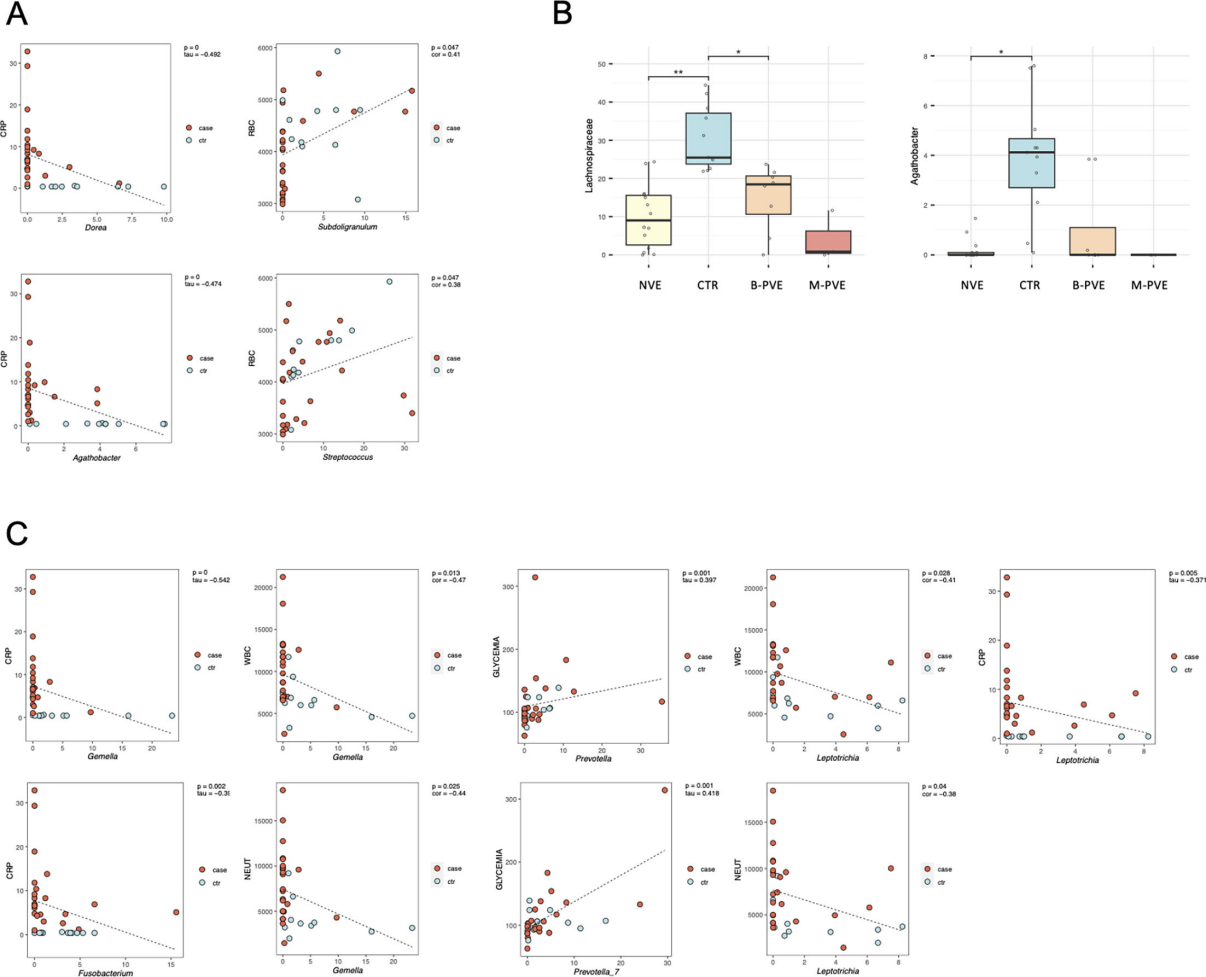
Associations between microbial genera and host clinical features**.** (**A**) Scatter plots showing correlations between gut microbiota genera and host metadata for cases and controls. (**B**) Boxplots showing the relative abundance of gut taxa differentially represented between cases and controls stratified by valve type (NVE: cases with native valve; CTR: controls with native valve; B-PVE: cases with bioprosthetic valve; M-PVE: cases with mechanical valve). Wilcoxon test, *P ≤ 0.05; **P ≤ 0.01. (**C**) Scatter plots showing correlations between oral microbiota genera and host metadata for cases and controls. Only statistically significant correlations (*P* ≤ 0.05) with absolute Kendall’s tau ≥ 0.3 for genera with relative abundance ≥1% are shown.

Regarding associations between bacterial taxa and patient clinical data, we found inverse correlations between C-reactive protein and both *Dorea* and *Agathobacter*, and direct correlations between red blood cell count and both *Subdoligranulum* and *Streptococcus* (Kendall correlation test, *P* ≤ 0.05) in the gut microbiota (Fig. 3A). Furthermore, *Agathobacter* and its family *Lachnospiraceae* were less represented in the gut microbiota of cases with native and bioprosthetic valves compared to controls (all with native valves) (Wilcoxon test, *P* ≤ 0.05) (Fig. 3B). Regarding the oral microbiota, we found several associations, including inverse correlations between *Fusobacterium* and C-reactive protein, between *Gemella* and *Leptotrichia* and several hematological parameters (i.e., C-reactive protein, white blood cell count, and neutrophils), and between *Prevotella* and *Prevotella 7* and glycemia (Kendall correlation test, *P* ≤ 0.05) (Fig. 3C).

### Oral, gut, and skin microbiota of patients with heart valve disease with infective endocarditis stratified by etiological agent and antibiotic treatment

Cases were then stratified by etiological agent (i.e., genus of microorganism isolated from blood cultures), and all analyses were repeated (Fig. S3). Overall, lower alpha diversity was found in cases compared to controls in all ecosystems, regardless of etiological agent (Wilcoxon test, *P* ≤ 0.05). Furthermore, all groups of cases still segregated from controls in the unweighted UniFrac-based PCoA for both oral and skin microbiota (PERMANOVA, *P* ≤ 0.05). A similar segregation was found for the gut microbiota, except for cases infected with *Enterococcus* strains. Taxonomically, some of the differences that emerged in the general cohort were replicated in some groups of cases, probably closely related to the etiological agent, whereas others were shared by all case groups, probably representing more universal dysbiotic features related to IE pathogenesis. In particular, the variation in Patescibacteria, *Gemella*, and *Neisseria* in oral microbiota, and that in *Coriobacteriaceae* (including its genus *Collinsella*), *Lachnospiraceae* (including *Agathobacter*, *Blautia*, and *Dorea*), and *Subdoligranulum* for gut microbiota, was confirmed irrespective of etiological agent (Wilcoxon test, *P* ≤ 0.05). In contrast, none of the above-mentioned variations for skin microbiota were common to all case groups. Regarding the genera of microorganisms isolated from blood cultures of IE patients, *Enterococcus* was found to be actually increased in the gut microbiota of cases infected with *Enterococcus* strains, while *Staphylococcus* was increased in the skin microbiota of cases infected with *Enterococcus* strains compared to cases infected with *Streptococcus* strains (*P* ≤ 0.05).

Similarly, when stratifying cases by antibiotic use (no use vs more than three days), we found that some differences observed compared with controls in the general cohort appeared only in treated cases, while others were observed in both treated and untreated cases, potentially representing antibiotic-driven and antibiotic-independent dysbiotic features of IE, respectively (Fig. S4 and S5). In particular, the variation in *Neisseria* in oral microbiota, and that in *Bacteroidaceae* (including *Bacteroides*), *Erysipelatoclostridiaceae*, *Lachnospiraceae* (including *Agathobacter* and *Blautia*), and *Subdoligranulum* for gut microbiota, was confirmed in both antibiotic-treated and non-treated cases compared with controls (Wilcoxon test, *P* ≤ 0.05). On the other hand, the variation in Fusobacteriota (including *Fusobacteriaceae* and *Fusobacterium*), *Gemellaceae* (including *Gemella*), *Porphyromonadaceae* (including *Porphyromonas*), *Haemophilus*, *Leptotrichia*, and *Alloprevotella* for oral microbiota, and that in *Dorea* for gut microbiota, was closely related to antibiotic use, being significant only in treated cases compared to both untreated cases and controls (*P* ≤ 0.05). For other antibiotic-driven changes, see Fig. S5. No differences were found in the skin microbiota in relation to antibiotic use. Taken together, these observations emphasize the important confounding effect of antibiotics and suggest that some of the dysbiotic features associated with IE may, at least in part, be influenced by treatment.

## DISCUSSION

IE is largely caused by a limited number of bacterial species, generally part of the host’s commensal microbiota, and often has a spontaneous onset. Therefore, the hypothesis that changes in the host microbiota might favor or have pathogenetic relevance in IE is potentially realistic. In our pilot study, by in-depth assessment of the microbiota in IE patients and valve disease controls, we showed that microbiota alterations are present in the mouth, gut, and skin of IE patients and could play a potential pathophysiological role.

In its early phase, IE often has a non-specific presentation, with fever being the main symptom. For this reason, it is very common for patients to be diagnosed with IE after one or more empiric antibiotic courses, which could affect the microbiota. As a matter of fact, it was very difficult to find IE patients who had not already received antibiotics at enrollment. In our cohort, 19 patients were on antibiotics prior to enrollment, with a high variability in the type of molecules administered, their combination, and the duration of previous therapy, which did not allow a detailed analysis of their specific effects on the microbiota. Nevertheless, all patients were taking at least one broad-spectrum antibiotic (such as beta-lactams or cephalosporins) or a combination of molecules active against both Gram-positive and Gram-negative bacteria, which is very likely to have influenced the composition of their microbiota. The widespread use of antibiotics prior to enrollment is an important confounding factor that must be considered when interpreting the results. Some of the dysbiotic features in cases that received antibiotic treatment may actually reflect the effects of the treatment rather than changes specific to IE, highlighting the difficulty of distinguishing disease-associated effects from those driven by antibiotics.

According to our findings, cases differed from controls in alpha and beta diversity and compositional structure in almost all microbial ecosystems considered. In particular, the oral and skin microbiota of cases showed lower diversity than controls, which is a typical non-disease-specific feature of dysbiosis (31). Taxonomically, the oral microbiota of cases was characterized by the depletion of many core commensals, such as *Gemella*, *Neisseria*, *Haemophilus*, *Corynebacterium*, *Rothia*, *Porphyromonas*, *Fusobacterium*, *Leptotrichia*, and *Streptococcus* (32–34), indicating a general impoverishment of the ecosystem, probably no longer able to maintain a symbiotic relationship with the host when IE develops. On the other hand, the oral cavity of the cases showed an enrichment in *Oxalobacteraceae*, a family for which little information is available but is generally associated with adverse health outcomes, especially in early life (21). It should be noted that some of the above variations (namely those in *Gemella*, *Haemophilus*, *Porphyromonas*, *Fusobacterium*, and *Leptotrichia*) were significant only in antibiotic-treated cases compared to both untreated cases and controls, most likely representing antibiotic-induced perturbations less closely related to IE onset. Similarly, the gut microbiota of cases was depleted of typical dominant taxa, such as those belonging to the core health-associated families *Lachnospiraceae* and *Ruminococcaceae*, and enriched in *Bacteroides*. Again, the decrease in beneficial commensals is a known dysbiotic feature common to multiple diseases, both local and systemic (31). Fewer and smaller changes were observed in the skin microbiota, including a reduction in common residents, such as *Moraxellaceae* and *Comamonadaceae* (35, 36).

Another point of interest in our analysis concerned the possible relation between IE etiological agents and the dysbiotic features we found. Literature on this specific topic is unfortunately still scarce. It is useful to remind that a necessary but not sufficient condition to develop IE is bacteremia, the penetration of microorganisms into the bloodstream through different physical barriers. Dysbiosis could facilitate this step, but not every dysbiotic environment provides barriers to lesions. When specifically searching for the genera of microorganisms isolated from the blood cultures of the cases, we found that *Enterococcus* was indeed increased in the gut microbiota of cases infected with *Enterococcus* strains. Furthermore, patients with enterococcal IE had a significantly lower diversity of gut microbiota, suggesting that this feature may be associated with increased susceptibility to the disease. In contrast, no significant match was found for *Staphylococcus* and *Streptococcus* in any of the ecosystems, while we found an apparently counterintuitive prevalence of *Streptococcus* in the skin microbiota of all cases, and overabundance of *Staphylococcus* in the skin microbiota of cases infected with *Enterococcus* strains. Furthermore, the consistent presence of *S. gallolyticus* in the host microbiota of patients with IE caused by this pathogen is particularly relevant. In line with the available literature (37, 38), we confirmed that *S. gallolyticus* also resides in the oral cavity, suggesting a possible additional source of bacteremia other than colorectal disease. On the other hand, some bacteria, such as *S. aureus*, have evolved the ability to overcome physical, immune, and chemical host defenses, allowing them to integrate into the host microbiome in different niches, disrupt its homeostasis, and take advantage of host defense leakages to become invasive (39). Overall, these results suggest a complex interplay between host microbiota, antibiotic exposure, and IE and highlight the potential value of future microbiota-targeted preventive or therapeutic strategies in high-risk patients.

Finally, our data showed specific associations between microbiota profiles and host response to infection, such as the inverse association between some core oral commensals (*Gemella*, *Fusobacterium*, *Leptotrichia*) and inflammatory markers, such as C-reactive protein, white blood cell count, and neutrophils, suggesting that the dysbiotic environment could affect the pro-inflammatory state and some illness features.

Although no definitive conclusions can be drawn, as dysbiosis may be associated with the risk or prognosis of IE, our findings could pave the way for the development of microbiota-targeted preventive or therapeutic strategies in patients with established risk factors for IE (i.e., valve defects, presence of prosthesis, or prior IE).

### Study limitations

The main limitations of this study include: (i) the relatively small sample size, which reduces the statistical power (particularly for any subgroup analyses) and generalizability of the findings, although IE is an uncommon disease and patients very often present at tertiary care centers after receiving prolonged antibiotic treatment; (ii) the lack of a true reference group, as controls had non-infectious valve disease and may exhibit microbiota dysbiosis themselves, potentially making the observed differences more conservative; (iii) the lack of information on major microbiota-associated confounding factors, such as dietary habits and lifestyles, which could influence alpha and beta diversity, as well as differential abundance patterns; (iv) the combined use of antibiotics from different classes, which biases the overall findings and limits the ability to assess the effects of specific antibiotics on the microbiota; (v) the use of 16S rRNA amplicon sequencing, which remains the gold standard for microbiota profiling but does not provide strain-level resolution, particularly relevant for diseases involving potential microbial exchange between different body sites, such as IE; (vi) the lack of extraction and library blanks, which are useful for controlling contamination in low-biomass samples, such as skin swabs; however, our data are consistent with the available literature, and no-template controls routinely performed in our laboratory using the same reagents and workflow conditions do not consistently reveal any detectable contamination or “kitome” signatures, suggesting that contamination is unlikely to have affected our results; and (vii) the use of non-parametric tests for microbiota data, which carry a risk of false positives in compositional data; however, *P*-values were corrected using the Benjamini-Hochberg method, and beta diversity differences were evaluated using PERMANOVA, which assesses overall community differences rather than individual taxa, partially mitigating the compositionality issue.

### Conclusions

By simultaneously examining the microbiota from three major niches of the human body, this observational pilot study identified dysbiotic features in the mouth, gut, and skin of patients with heart valve disease with IE compared to those without IE, including lower diversity in oral and skin communities and overall community depletion. Only a few bacteria were enriched, such as *Oxalobacteraceae* in the oral cavity and *Bacteroides* in the gut, which deserve further study at a low taxonomic level to explore their potential role in IE, including underlying mechanisms. Furthermore, a low concordance was found between bacterial changes and blood isolates for most IE-causing bacteria, suggesting that dysbiosis may have effects beyond merely acting as a reservoir for specific pathogens. However, given the exploratory nature of the study and its main limitations, including the confounding effect of antibiotics, all findings—particularly those related to subgroup analyses—should be treated with caution and considered as generating hypotheses rather than providing definitive answers. Further research is needed to validate and extend these observations, elucidating the confounding effect of antibiotics and unraveling potential pathogenetic mechanisms. This research should include larger longitudinal cohorts, possibly including antibiotic-naïve patients, as well as *in vitro* and *ex vivo* studies.

## Data Availability

All data generated or analyzed during this study are included in this published article

## SUPPLEMENTAL MATERIAL

Supplemental Material spectrum.01908-25-s0001.docx Figures S1 to S5; Table S1.

ASM does not own the copyrights to Supplemental Material that may be linked to, or accessed through, an article. The authors have granted ASM a non-exclusive, world-wide license to publish the Supplemental Material files. Please contact the corresponding author directly for reuse.
