# Supplementary material for: Oral, gut, and skin microbiota characterization in patients with heart valve disease with or without infective endocarditis: a pilot study
Source: Microbiol Spectr. 2026 Jul 17;14(8):e01908-25. doi: 10.1128/spectrum.01908-25 (PMC13436146; doi:10.1128/spectrum.01908-25)
Supplement: Supplementary file 1 [file spectrum.01908-25-s0001.docx]

**A**

**B**

**C**

**Supplementary Figure 1. Phylum-, family- and genus-level composition of the oral, gut and skin microbiota in patients with heart valve disease with and without infective endocarditis.** Bar plots (top) and pie charts (bottom) showing the relative abundance profiles of phyla (A), families (B) and genera (C) of the oral, gut and skin microbiota of cases and controls. Only taxa with relative abundance ≥1% in at least 20% of samples for each study group are shown; those with relative abundance <1% were grouped as “Other”.

**Supplementary Figure 2. Oral, gut and skin microbiota signatures of patients with heart valve disease with or without infective endocarditis.** Boxplots showing the relative abundance of phyla (A), families (B) and genera (C) differentially represented in the oral, gut and skin microbiota of cases and controls. Wilcoxon test, * p≤0.05; ** p≤0.01.

**Supplementary Figure 3. Oral, gut and skin microbiota of patients with heart valve disease and infective endocarditis, stratified by aetiological agent, compared to controls.** A, Boxplots showing alpha diversity, estimated using Faith’s phylogenetic diversity (PD whole tree), the number of observed ASVs and the Shannon index, in the oral, gut and skin microbiota of controls and cases, the latter stratified by genus of microorganism isolated from blood cultures (*Staphylococcus*, *Streptococcus* and *Enterococcus*). Wilcoxon test, * p≤0.05; ** p≤0.01. B, Principal Coordinates Analysis (PCoA) based on unweighted UniFrac distances between the microbial profiles of the study groups. A significant separation between all case groups and controls was observed for both oral and skin microbiota (PERMANOVA, p≤0.05). A similar segregation was found for the gut microbiota, except for cases infected with *Enterococcus* species. Boxplots showing the relative abundance of taxa differentially represented in the oral (C) and gut (D) microbiota of the study groups. Only taxa that increased or decreased in all case groups (regardless of aetiological agent) compared to controls are shown, along with *Enterococcus*. Wilcoxon test, * p≤0.05; ** p≤0.01.

**Supplementary Figure 4. Antibiotic-independent dysbiotic features of infective endocarditis in the oral and gut microbiota of patients with heart valve disease.** Boxplots showing the relative abundance of taxa differentially represented in the oral (A) and gut (B) microbiota of controls and cases, the latter stratified by antibiotic use (no vs. yes, more than 3 days). Only taxa that increased or decreased in both case groups (regardless of antibiotic use) compared to controls are shown. Wilcoxon test, * p≤0.05; ** p≤0.01.

**Supplementary Figure 5. Antibiotic-driven dysbiotic features of infective endocarditis in the oral and gut microbiota of patients with heart valve disease.** Boxplots showing the relative abundance of taxa (top, phyla; centre, families; bottom, genera) differentially represented in the oral (A) and gut (B) microbiota of controls and cases, the latter stratified by antibiotic use (no vs. yes, more than 3 days). Only taxa that increased or decreased in treated cases compared to untreated and/or control groups are shown. Wilcoxon test, * p≤0.05; ** p≤0.01.

**Supplementary Table 1. relative abundance data for differentially represented taxa.** For each differentially represented taxon (at phylum, family, and genus level) in the oral, gut, and skin microbiota of patients awaiting heart valve replacement in the presence (cases) or absence (controls) of infective endocarditis, the mean relative abundance in each study group and the direction of change (↑ increased or ↓ decreased in cases) are reported. Please, see Figure 2 and Supplementary Figures 1 and 2.
